# The Self‐Management Assessment Scale: Development and psychometric testing of a screening instrument for person‐centred guidance and self‐management support

**DOI:** 10.1002/nop2.233

**Published:** 2018-12-26

**Authors:** Ulrika Öberg, Åsa Hörnsten, Ulf Isaksson

**Affiliations:** ^1^ Department of Nursing Umeå University Umeå Sweden

**Keywords:** instrument development, nursing, person‐centred care, psychometric properties, self‐management support, type 2 diabetes

## Abstract

**Aim:**

To develop and psychometrically test the Self‐Management Assessment Scale (SMASc), a screening instrument for person‐centred guidance and self‐management support of persons with type 2 diabetes (T2D).

**Background:**

T2D is a common and globally increasing chronic condition. Improved self‐management is a vital and integral component of diabetes care to prevent complications from poorly managed diabetes. For diabetes nurses to better understand persons with diabetes experienced challenges and needs regarding self‐management and further for persons with T2D to take an active role in managing their condition, an instrument measuring this is needed.

**Design:**

Instrument development and psychometric testing of the content and construct validity, factor structure and reliability.

**Method:**

The SMASc was psychometric tested on a sample of participants (September 2017–November 2017) with a confirmed diagnosis of T2D (*N* = 104).

**Results:**

Psychometric findings were satisfactory and supported the scale´s reliability. Cronbach's alpha, CVI and goodness‐of‐fit were acceptable.

**Conclusion:**

Self‐Management Assessment Scale is a short validated screening instrument, which can indicate possible barriers for self‐management that ought to be approached during the conversation between the person with T2D and the primary healthcare nurses. Therefore, it is a promising instrument to be used to facilitate person‐centred guidance and to improve self‐management of people living with T2D.

## 1 INTRODUCTION

Type 2 diabetes mellitus (T2D) is a progressive chronic condition presenting with hyperglycaemia (ADA, 2015), and its prevalence is increasing globally (Ogurtsova et al., 2017). The emphasis of treatment is on self‐management support and education, which is recognized as an important component for the management of T2D and aims at especially controlling blood glucose and postponing severe disease complications (Inzucchi et al., 2015; Powers et al., 2015).

However, effective self‐management in daily life can be a challenge to many people living with chronic conditions, as T2D. People living with T2D, with a variety of complex self‐management activities and skills as self‐monitoring of blood glucose, medication use, foot exams, physical activities and health eating (Powers et al., 2015), must be able to identify and correct problems when it occurs due to the disease (Jutterström, Hällgren‐Graneheim, Isaksson, & Hörnsten, 2012).

Since the patients have different individual needs and goals, a challenge for the diabetes nurse is to discuss with each patient, what different personalized education and support are needed on how to manage just their T2D (Hörnsten, Stenlund, Lundman, & Sandström, 2008; Jutterström, Hällgren‐Graneheim, et al., 2012; Munshi et al., 2016). With a person‐centred approach and tailored education and a collaboration/partnership between patients and healthcare professionals striving towards the same goals (Graneheim ‐ Hällgren & Hörnsten, 2011; Hörnsten, Lundman, Selstam, & Sandström, 2005), the patient's empowerment can be strengthened (Rossi et al., 2015).

For diabetes nurses to better understand persons with T2D and their experienced challenges and personal needs regarding self‐management and further for them to take an active role in managing their condition, an instrument that measures this is needed.

This study attempts to contribute by developing and psychometrically test the Self‐Management Assessment Scale (SMASc), a screening instrument for person‐centred guidance and self‐management support of persons with type 2 diabetes (T2D).

## 2 BACKGROUND

Society worldwide as well as in Sweden faces dramatic challenges with a changed composition of the population (WHO, 2015), implying an increased amount of older (Statistics Sweden, 2017; WHO, 2015), chronically ill and multi diseased people. Meeting their care needs poses also important economic and social challenges (Alwan, Armstrong, Cowan, & Riley, 2011; National Board of Health & Welfare, 2016; WHO, 2013; WHO, 2015; WHO, 2016). With limited healthcare resources, health care needs to develop and become more effective, simultaneously maintain and ensure high‐quality care. In addition, evidence‐based medicine, person‐centred care and eHealth are promoted from policymakers. This is an equation with no easy solution.

Type 2 diabetes mellitus is a common and globally increasing chronic condition, which besides heritage is related to lifestyle risk factors, such as inactivity and obesity (Franz, Zhang, & Venn, 2017; Hu, 2011; Ogurtsova et al., 2017). If not treated effectively, T2D can lead to severe complications such as cardiovascular disease and neuropathy (ADA, 2016).

Improved self‐management is a vital and integral component of diabetes care to prevent complications from poorly managed diabetes. Self‐management in T2D implies managing symptoms and impairments, following medical regimes, pursuing interactions with the health care and not least performing lifestyle changes including exercise, healthy eating, weight management, foot exams and self‐monitoring blood glucose (ADA, 2016; Bodenheimer, Lorig, Holman, & Grumbach, 2002; Disler, Gallagher, & Davidson, 2012; Powers et al., 2015). Several authors highlight that self‐management must include acquiring knowledge, abilities and skills around the management of chronic illness (Barlow, Wright, Sheasby, Turner, & Hainsworth, 2002; Chodosh et al., 2005; Corbin & Strauss, 1988). The change from a provider‐centred to person‐centred care has been described as a paradigm shift, where patients' autonomy and accountability are strengthened (Ekman et al., 2012). To provide person‐centred care, it is underlined the importance of flexibility among healthcare staff to best adapt routines based on patients and their needs (Edvardsson, 2015).

Primary healthcare nurses/diabetes nurses in Sweden play an important key role in guiding and supporting persons in their self‐management efforts. In previous studies, it have been reported that primary healthcare nurses seem to be ambivalent towards person‐centred care (Boström, Isaksson, Lundman, Graneheim‐Hällgren, & Hörnsten, 2014). Boström, Isaksson, Lundman, Lehuluante, & Hörnsten, 2014) and have difficulties to know which area they should focus on in self‐management support (Hörnsten et al., 2008; Jutterström, Hällgren‐Graneheim, et al., 2012). Often, the same information is given to everyone despite different needs. This is something that persons with T2D have described as problematic, that is not striving towards the same goals (Graneheim ‐ Hällgren & Hörnsten, 2011; Hörnsten et al., 2005). Therefore, nurses responsible for T2D care seem to require a tool to more effectively screen self‐management needs.

ADA highlights the importance of using self‐management instruments to visualize strengths and weaknesses and states that psychosocial issues are lacking in existing instruments and, therefore, should be included in forthcoming instrument development (cf. Marathe, Gao, & Close, 2017). Consequently, we intended to develop a screening instrument measuring persons’ needs for self‐management support in various areas. The instrument should be person‐centred, practical and applicable for use among nurses in primary health care and preferably facilitate guidance of people with T2D regarding self‐management.

### 2.1 The study

#### 2.1.1 Aim

The aim of this paper was to develop and psychometrically test the Self‐Management Assessment Scale (SMASc), a screening instrument for person‐centred guidance and self‐management support of persons with type 2 diabetes (T2D).

## 3 METHODOLOGY

The SMASc instrument was developed in several steps and psychometrically tested with different methods (Streiner & Norman, 2008) presented in Table 1.

**Table 1:** Overview of the development and validation process

	Method	Sample	Result
Phase I: Instrument development			
1. Decision about theoretical framework	Inventory of the literature (review) and reflections from an expert panel	Expert panel (*N* = 5)	Self‐management, person‐centred care, illness integration, self‐efficacy, health literacy
2. Categorization of concepts related to self‐management	Brainstorm and categorization of content	Expert (*N* = 7), patients with T2D (*N* = 3)	Seven domains were created labelled: knowledge, routines, will, decision‐making, planning, social support and emotions
3. Scale construction			28 items in seven domains were constructed with two positive and two negative items/domain scored at a six‐point response option
4. Item analysis	Calculation of CVI Evaluation of face‐ and content validity	Expert panel (*N* = 5) A convenience sample of teachers (*N* = 88)	Average CVI for total scale = 0.89 Some items were reworded
5. Instrument refinement	Reliability test and item reduction Distribution and skewness of responses per item EFA Cronbach's *α*	Patients with chronic illness, including T2D (*N* = 138)	Negative and cross‐loaded items were deleted, resulting in totally ten items distributed in five domains, reformulated to knowledge, goals for future, daily routines, emotional adjustment and social support
Phase II: Psychometric analysis		Adults with T2D from four primary healthcare centres (*N* = 104)	
1. Item distribution	Distribution and skewness of responses per item		Skewness in four items. Missing values low
2. Construct validity	Parallel analysis EFA CFA		Recommended number of factors = 1 Insufficient concordance with a five‐factor model Good model‐of‐fit
3. Internal reliability	Cronbach's *α*		Cronbach's alpha was analysed for total scale and domains and found satisfactory

Note

CFA: Confirmatory factor analysis; CV: Content validity index; EFA: Exploratory factor analysis.

### 3.1 Phase I: Instrument development

Members in the research group with experience on personal understandings (Hörnsten, 2004), illness integration (Jutterström, 2013) and person‐centred care in T2D (Boström, 2013) discussed and came to a consensus about the theoretical framework for the scale development. An inventory of the literature was performed about patient perspectives on self‐management and living with T2D (Hörnsten, Jutterström, Audulv, & Lundman, 2011; Isaksson, Hajdarevic, Abramsson, Stenvall, & Hörnsten, 2015; Jutterström, Isaksson, Sandström, & Hörnsten, 2012; Kralik, Koch, Price, & Howard, 2004; Whittemore, Chase, Mandle, & Roy, 2002; Whittemore & Dixon, 2008) and integrated into a concept base of important themes related to self‐management in T2D.

Seven researchers and three patients performed a 777 brainstorm session to highlight and categorize important concepts related to self‐management. Words or concepts were written on a whiteboard and discussed concerning content and relevance. These words and concepts were then categorized into seven domains labelled: knowledge, routines, will, decision‐making, planning, social support and emotions.

Two positive and two negative items/statements were constructed for each domain, resulting in an initial 28 items instrument. A six‐point response option was chosen, ranging from 1 (Strongly disagree) to 6 (Totally agree). The reason for using a six‐point scale was to get variability in the answers and obtain reliability (Streiner & Norman, 2008).

As an item analysis, content validity was evaluated using content validity index (CVI; Polit & Beck, 2006). To evaluate the 28 items, five researchers, one professor emerita, two professors and two senior lecturers with expertise in patient perspectives of various chronic conditions were involved whereby minor rewordings were made. The average CVI for the total scale was 0.89. The 28‐item instrument was also validated in a convenient sample of teachers (*N* = 88) resulting in further minor rewording.

To refine the instrument, patients (*N* = 138) from primary health care and hospital‐based diabetes clinics were recruited to fill in the 28‐item instrument. Statistical analyses including descriptive statistics and reliability estimates were performed. Each item was analysed concerning distribution and skewness of responses. Explanatory factor analysis of the initial seven‐domain instrument was performed. Cronbach's alpha was analysed for total scale and domains. The analysis showed that the item in the different domains cross‐loaded, especially among the negative items. This led to an instrument refinement where negative items and cross‐loaded items were deleted, resulting in a 10‐item instrument distributed in five domains with two items each.

### 3.2 The SMASc instrument

The current instrument assesses five domains important for effective self‐management over time: knowledge, goals for future, daily routines, emotional adjustment and social support (Table 2). The background and related references strengthening the relevance for self‐management are explained more in detail below.

**Table 2:** The final SMASc instrument

	Strongly disagree				Totally agree	
I have enough knowledge about my condition	1	2	3	4	5	6
I have good social support, which makes it easier for me	1	2	3	4	5	6
I have those who support me to make self‐management work	1	2	3	4	5	6
I find joy in everyday life despite my illness	1	2	3	4	5	6
I know how to handle the illness in daily life	1	2	3	4	5	6
I have found good daily routines	1	2	3	4	5	6
I have received sufficient amount of information	1	2	3	4	5	6
I feel satisfied with my situation	1	2	3	4	5	6
I have a plan for how to deal with my illness	1	2	3	4	5	6
I have concrete plans for my future self‐management	1	2	3	4	5	6

*Knowledge* concerns facts and information as a part of informational health literacy (Batterham, Hawkins, Collins, Buchbinder, & Osborne, 2016; Sørensen et al., 2015). For example, it assesses needs to get more knowledge about the disease, bodily functions and/or medication, diet or exercise alternatively illness management. Furthermore, contact information for healthcare units, professionals and social—as well as patients—or voluntary organizations are examples of knowledge needs important for people with chronic illness.

*Goals for future* concern goals and plans for daily life. Having a concrete goal for self‐management activities is more beneficial than general, non‐specified plan. Not having any plans or not even having a will to make a plan could be an issue about not being prepared for change (Bratzke et al., 2015; Fitzpatrick et al., 2016; Hoffmann et al., 2014). Future plans relate to coping (Hajdarevic, Schmitt‐Egenolf, Sundbom, Isaksson, & Hörnsten, 2013) as well as needs to integrate illness and self‐management in life (Jutterström, 2013), for example, practical activities such as self‐monitoring, taking medication, adjusting diet and exercise if needed.

*Daily routines* concern how to handle illness smoothly in daily life. It involves the development of patterns such as for exercise and diet. It may also involve readiness for change, during travels and other new circumstances. An insight into a need of changed daily routines is commonly not enough to initiate a new behaviour (Jutterström, 2013). Getting help from a nurse, a dietician or a physiotherapist to schedule and evaluate self‐management activities could be one way to create daily routines, which also could strengthen self‐efficacy (Abubakari, Cousins, Thomas, Sharma, & Naderali, 2016; Aljasem, Peyrot, Wissow, & Rubin, 2001) and illness integration (Boström, 2013; Hernandez, 1996; Jutterström, 2013).

*Emotional adjustment* concerns the emotional and existential process a person, who is diagnosed with a long‐term illness, has to pass to experience normality (Jutterström, 2013) and satisfaction. Emotional adjustment commonly involves the identity and role change. It also concerns dealing with threats such as fear of complications or even death implied with the long‐term disease. Fear instead of life satisfaction could be an obstacle for rational decision‐making around self‐management. Too little emotional reaction though could also be an obstacle for self‐management (Hörnsten et al., 2011).

*Social support* concerns the importance of strengthening social networks, which facilitates self‐management (Ericson‐Lidman, 2008). Family, friends and workmates could be involved at various levels. Patient organizations and other voluntary organizations are other examples. Family members and social circumstances may also be obstacles for self‐management (Koetsenruijter et al., 2016). Even healthcare professionals have in studies been described as either supportive or counteracting self‐management or illness integration (Boström, Isaksson, Lundman, Graneheim‐Hällgren, et al., 2014; Hörnsten et al., 2005).

### 3.3 Scoring of SMASc

**Figure 1:**
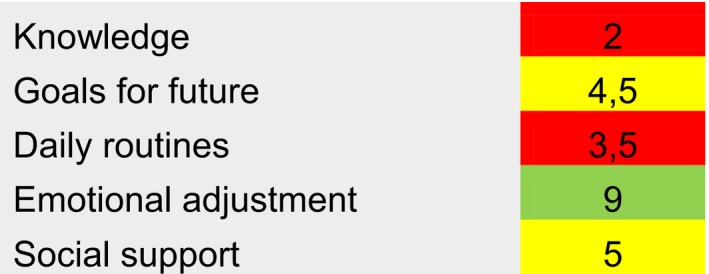
Example of interpretation of SMASc

Each item is scored from 1 (Strongly disagree) – 6 (Totally agree), using a six‐point Likert scale. Each domain is ranging from 2 – 12 where low score represents higher needs of self‐management support. The instrument cannot calculate a total self‐management assessment score. However, for each domain, a mean score is calculated as follows: Knowledge = added scores on items 1 + 7, Goals for future = 9 + 10, Daily routines = 5 + 6, Emotional adjustment = 4 + 8 and, finally, Social support = 2 + 3. For each domain, mean score 1–4 is interpreted as “Immediate need for self‐management support,” a score 5–8 is interpreted as “No acute need for self‐management support,” and finally, a score 9–12 is interpreted as “No need for self‐management support.” These scores are categorized and can be presented in a graphic profile. An example of interpretation is given in Figure 1.

### 3.4 Data analysis

Content validity on item level (CVI) was assessed by a panel of five experts, who rated each scale item regarding its relevance to the underlying construct. We then computed the percentage of items deemed to be relevant for each expert and took an average of the percentages across experts (Polit & Hungler, 1999). According to Polit and Hungler (1999), content validity is “the degree to which the items in an instrument adequately represent the universe of content.”

The psychometric analysis included an assessment of the items’ distributional properties (i.e., distribution in per cent and skewness for each item). A parallel analysis (PA) was performed to determine the number of common factors in the instrument. Exploratory factor analysis (EFA) was performed to explore the underlying structure of the 10‐item SMASc, while confirmatory factor analysis (CFA) was performed to evaluate the model fit.

During EFA, the ten items were forced into a five‐factor solution based on the theoretical model, using principal axis factoring as the extraction method with direct oblique rotation (Costello & Osborne, 2005). The extraction method was chosen because the data were not assumed to be normally distributed; direct oblimin rotation was chosen because the factors were expected to correlate with each other.

A secondary EFA with eigenvalues over 1 was performed. A confirmatory factor analysis was performed to assess goodness‐of‐fit by means of chi‐square (*χ* ^2^) and chi‐square/degrees of freedom (*χ* ^2^/*df*), Tucker–Lewis index (TLI), normed fit index (NFI), comparative fit index (CFI) and root mean square error of approximation (RMSEA). Internal consistency was estimated by Cronbach's alpha.

The SPSS version 23.0 (SPSS Inc., Chicago, IL, USA) and AMOS version 23.0 (SPSS Inc.) statistical software packages were used for statistical analysis. Parallel analysis was performed using FACTOR (Universitat Rovira i Virgili, Tarragona, Spain) version 8.02.

### 3.5 Ethical considerations

According to the Swedish Act on Ethics (SFS, 2003), this analysis of the psychometrics of the instrument does not require ethical approval. The participants included for the psychometric analysis were informed about the study, requested in person and informed that they could withdraw without giving any reason and were assured confidentiality, and no personal data were collected.

## 4 RESULTS

### 4.1 Phase II: Psychometric analysis

#### 4.1.1 Sample and setting

For testing the SMASc instrument, a consecutive sample of adults with diabetes was recruited from four primary healthcare centres in northern Sweden. Inclusion criteria were as follows: confirmed diagnosis of diabetes and ≥18 years old. A sample of 104 participants with a mean age of 50.22 years (SD 18.90) (53 men, 51 women) was enrolled in this study.

##### Item distribution

An initial analysis of the distribution of responses showed skewness in four items. Six items (2, 3, 6, 8, 9, & 10) were considered to be evenly distributed. However, the number of missing items was very low (Table 3).

**Table 3:** Distribution, skewness and kurtosis of responses per item. Swedish items in italics

	Items	Md	1	2	3	4	5	6	Mis	Sk	Ku
1	I have enough knowledge about my condition *Jag har tillräcklig kunskap om mitt tillstånd*	5	1.9	2.9	10.6	11.5	49.0	23.1	1.0	−1.22	1.41
2	I have good social support, which makes it easier for me *Jag har ett gott socialt stöd vilket underlättar för mig*	5	2.9	7.7	10.6	17.3	25.0	36.5	—	−0.86	−0.20
3	I have those who support me to make self‐management work *Jag har dem som stöttar mig för att egenvården ska fungera*	5	2.9	7.7	12.5	13.5	27.9	35.6	—	−0.86	−0.26
4	I find joy in everyday life despite my illness *Jag finner glädje i vardagen trots min sjukdom*	5	3.8	2.9	9.6	8.7	33.7	41.3	—	−1.37	1.30
5	I know how to handle the illness in daily life *Jag vet hur jag skall hantera sjukdomen i vardagen*	5	1.9	4.8	7.7	12.5	47.1	26.0	—	−1.26	1.37
6	I have found good daily routines *Jag har hittat bra dagliga rutiner*	5	2.9	4.8	14.4	12.5	47.1	18.3	—	−0.99	0.45
7	I have received sufficient amount of information *Jag har fått information i tillräcklig utsträckning*	5	4.8	4.8	4.8	19.2	39.4	26.9	—	−1.25	1.17
8	I feel satisfied with my situation *Jag känner mig tillfreds med min situation*	4	7.7	5.8	12.5	25.0	34.6	14.4	—	−0.79	−0.03
9	I have a plan for how to deal with my illness *Jag har en plan för hur jag skall hantera min sjukdom*	5	2.9	6.7	12.5	22.1	28.8	26.0	1.0	−0.71	−0.19
10	I have concrete plans for my future self‐management *Jag har konkreta planer för min framtida sjukdomshantering*	4	7.7	10.6	11.5	22.1	32.7	14.4	1.0	−0.63	−0.55

##### Construct validity

**Figure 2:**
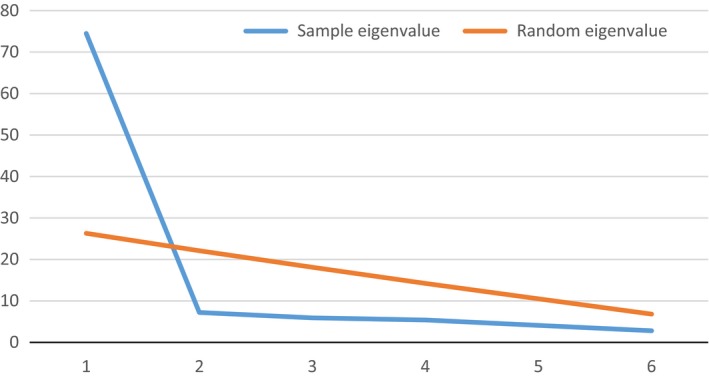
Scree plot of parallel analysis of the SMASc

Five experts in the field assessed content validity. The CVI for the SMASc was 0.89. A parallel analysis based on minimum rank factor analysis (Timmerman & Lorenzo‐Seva, 2011) was carried out to determine the minimum number of factors in the instrument. The result showed that the recommended number of factors was one (Figure 2). An initial exploratory factor analysis (EFA) showed that items with an eigenvalue over 1 loaded in only one domain. Therefore, a second EFA was performed which showed insufficient concordance with the purposed five‐factor model with one factor only one item loading. Furthermore, two items cross‐loaded in two different domains.

**Figure 3:**
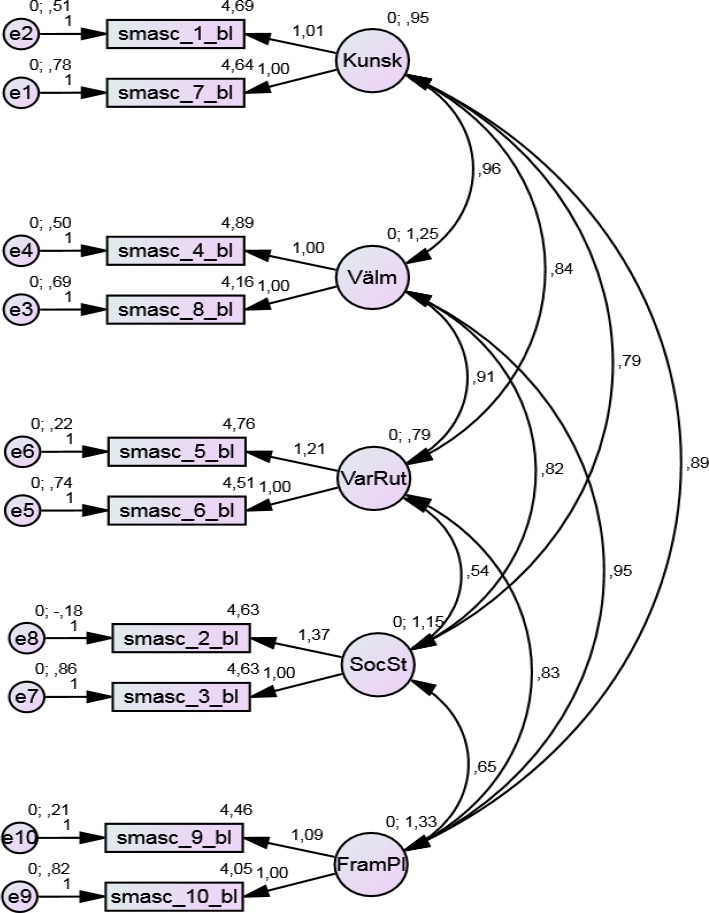
Model of the 10‐item version of the SMASc‐questionnaire with standardized regression weights and correlations

A confirmatory factor analysis (CFA) was performed on the five‐factor solution to test its goodness‐of‐fit. The chi‐square was significant (*χ* ^2^ = 46.0, *df* = 25, *p* < 0.006) with a relative chi‐square of 1.84. The TLI was 0.93; NFI 0.94; CFI 0.97; and, finally, RMSEA was 0.09 indicating a good model‐of‐fit. The final model is shown in Figure 3, including path coefficients (standardized regression weights and correlations). Correlations between the factors were in the interval of 0.54–0.96, and the highest correlation was found between knowledge and emotional adjustment. All of the path coefficients were significant at a *p* < 0.001 level.

##### Internal reliability

Internal consistence was estimated by Cronbach's alpha for respective domain resulting in for knowledge, *α* = 0.727; goals for future, *α* = 0.848; daily routines, *α* = 0.794; emotional adjustment, *α* = 0.807; and, finally, social support, *α* = 0.883. Cronbach's alpha for the instrument as of whole was 0.925.

## 5 DISCUSSION

This study aimed to describe the development and psychometrically test the Self‐Management Assessment Scale (SMASc) and provides preliminary support for the validity, reliability and utility of the instrument. The content validity of the SMASc is supported by the fact that it was derived from our previous theoretically based work in illness integration, patient perspectives on self‐management and person‐centred care in T2D. The CVI for the SMASc was 0.89. This can be considered as acceptable since standard acceptability for CVI is 0.80. Polit and Beck (2006) though accept CVI as low as 0.70 while Waltz, Strickland, and Lenz (2010) argue that CVI should be 0.90 or higher. A conclusion is therefore that the wording of the items is satisfactory.

Some items had a skewed distribution. However, this was expected since people tend to rate their situation more positive than negative. Furthermore, most people with chronic illness are in general coping with their disease effectively. Another explanation though could be that the number of participants was few and it was easy to rate on one of the extremes.

The PA suggested a one‐factor solution. This is in line with a theoretical assumption that the five domains relate to effective self‐management. Hayton, Allen, and Scarpello (2004) argue that in some situations PA can under factor. One of these situations is when there is a high correlation between factors, as in this case. This was also demonstrated in the further analysis in where an EFA showed only one domain.

The goodness‐of‐fit (TLI, NFI, CFI and RMSEA) was more than acceptable. However, the chi‐square test was significant, which is not preferable but the relative chi‐square was 1.84, below recommended 2.0 (Tabachnick, & Fidell, 2006; Wheaton, Muthen, Alwin, & Summers, 1977).

The reliability, measured by Cronbach's alpha, was for the whole scale 0.925 with the different domains varying from 0.727 to 0.883, which indicates a high internal consistency.

We consider that the instrument can be used in clinical practice. It was found to be easy and quick for people to complete and showed relatively good results. However, one important issue concerning this instrument is the fact that the domains only consist of two items each. This is not optimal since Costello and Osborne (2005) recommend that a domain should consist of three items or more. Therefore, for use in research and further psychometric evaluation, a development of the instrument is desirable.

This screening instrument is created for measuring patients’ needs for self‐management support. SMASc is a tool that can help healthcare professionals to tailor person‐centred guidance and self‐management support of people with chronic illness, such as type 2 diabetes. Our striving was to develop a short screening instrument that is useful, easy and quick to complete in day‐to‐day care by professionals, particularly nurses, in primary healthcare settings. With the presentation of the patient's score, the nurse and the patient together can target the individual need for improvement in self‐management support. Based on results from the SMASc‐scoring, nurses could recommend information sites on the Internet as well as mobile applications facilitating daily routines.

### 5.1 Limitations

The sample was enrolled in one country, and the sample size, although adequate for the purpose of the study, was relatively limited. However, content validity was estimated by a panel of experts in the field, persons living with T2D were represented, and the patient characteristics are aligned with the global diabetes population. Another limitation is that test–retest was not made in this study. Further studies are needed investigating the applicability of the SMASc instrument to promote person‐centred guidance and self‐management support of persons with T2D. Further studies are also necessary to obtain a more precise cut‐off between the different support levels.

## 6 CONCLUSION

We strived to measure the level of needs of self‐management support of patients with T2D. The Self‐Management Assessment Scale (SMASc) has been developed and validated in this study. The instrument is deemed to be useful and enables to build profiles of persons with chronic conditions about their self‐management skills. We found the SMASc screening instrument valuable to identify person‐centred self‐management support to provide to people with T2D treated in primary health care. A short screening instrument, such as the SMASc, might be particularly useful in healthcare settings where time constraints often occur.

## CONFLICT OF INTEREST

No conflict of interest has been declared by the authors.

## AUTHOR CONTRIBUTIONS

UÖ, ÅH, UI: Substantial contributions to conception and design, or acquisition of data, or analysis and interpretation of data; manuscript drafting and critical revision of it for important intellectual content; final approval of the version to be published. Each author participated sufficiently in the work to take public responsibility for appropriate portions of the content and agreed to be accountable for all aspects of the work in ensuring that questions related to the accuracy or integrity of any part of the work are appropriately investigated and resolved.
